# Deep learning predicts *IDH1* mutation status in acute myeloid leukemia from bone marrow smears

**DOI:** 10.1038/s41698-026-01762-x

**Published:** 2026-10-29

**Authors:** Jan-Niklas Eckardt, Chethan Babu V. Reddy, Andrew Srisuwananukorn, Christian Pohlkamp, Maryam Schübel, Ishan Srivastava, Miriam Stoerk, Sebastian Riechert, Tim Schmittmann, Martin M. K. Schneider, Klaus H. Metzeler, Karsten Spiekermann, Markus Badstübner, Susann Winter, Katja Sockel, Frank Kroschinsky, Christoph Röllig, Martin Bornhäuser, Tobias Herold, Torsten Haferlach, Ann-Kathrin Eisfeld, Jan Moritz Middeke

**Affiliations:** 1 Department of Internal Medicine I, University Hospital Carl Gustav Carus, TUD Dresden University of Technology, Dresden, Germany; 2 Else Kröner Fresenius Center for Digital Health, TUD Dresden University of Technology, Dresden, Germany; 3 Mildred Scheel Early Career Center, National Center for Tumor Diseases (NCT/UCC) Dresden, Faculty of Medicine and University Hospital Carl Gustav Carus, TUD Dresden University of Technology, Dresden, Germany; 4 Cancilico GmbH, Dresden, Germany; 5 Division of Hematology, Department of Internal Medicine, The Ohio State University Comprehensive Cancer Center, Columbus, OH USA; 6 MLL Munich Leukemia Laboratory, Munich, Germany; 7 Department of Medicine III, University Hospital, LMU Munich, Munich, Germany; 8 Department of Hematology, Cellular Therapy, Hemostaseology and Infectious Diseases, University Hospital Leipzig, Leipzig, Germany; 9 German Cancer Consortium (DKTK), Partner Site Dresden, and German Cancer Research Center (DKFZ), Heidelberg, Germany; 10 National Center for Tumor Diseases (NCT), NCT//UCC Dresden, a partnership between DKFZ, Faculty of Medicine and University Hospital Carl Gustav Carus, TUD Dresden University of Technology, and Helmholtz-Zentrum Dresden-Rossendorf (HZDR), Dresden, Germany; 11 Department of Hematology, Oncology, and Stem-Cell Transplantation, Munich Hospital Bogenhausen, Munich Municipal Hospital Group, Munich, Germany

**Keywords:** Cancer, Computational biology and bioinformatics, Diseases, Oncology

## Abstract

Acute myeloid leukemia (AML) with *IDH1* mutations is amenable to targeted therapy, highlighting the need for reliable identification. We developed an attention-based multiple-instance learning model to predict *IDH1* mutation status using bone marrow smear whole-slide images from 436 AML patients across four centers in Germany and the US, achieving a mean single pooled ROC-AUC of 0.81. In top tile analysis, model predictions for *IDH1* were associated with immature leukemia phenotypes.

Acute myeloid leukemia (AML) is a genetically heterogeneous disease. The rise of next-generation sequencing (NGS) has unveiled numerous recurrently altered genes in AML, a minority of which are also treatable via targeted therapy^1^. Alterations of the *isocitrate dehydrogenase 1 (IDH1)* gene are recurrent events in AML, affecting approximately 8% of patients^2^. These alterations impair normal cellular metabolism, leading to an accumulation of the oncometabolite 2-hydroxyglutarate (2-HG) and affecting epigenetic regulation and cellular differentiation^3^. Importantly, *IDH1* alterations carry therapeutic significance, as the selective inhibitors ivosidenib and olutasidenib are FDA-approved for the treatment of relapsed or refractory AML, with ivosidenib also approved in the upfront setting^4,5^.

## Rationale for image-based prediction of *IDH1* mutation status

Deep learning-based computer vision - the capability of machine learning algorithms to effectively process image data for segmentation, object detection, and image classification tasks - currently reshapes medical imaging^6^. In hematologic microscopy, computer vision algorithms have not only proven capable of cell segmentation and classification in cytomorphology, for example, for the diagnosis of AML^7^, but also in identification of digital biomarkers for potential clinical decision-making. The latter constitute genotype-phenotype links, i.e., visual cues in bone marrow morphology that are identified by computer vision algorithms to inform about underlying genetic alterations solely based on image data. In AML, such visual digital biomarkers have so far been identified for alterations of *NPM1*, *FLT3*-ITD, CBFB::MYH11 fusions or RUNX1::RUNX1T1 fusions, and t(15;17)/PML-RARa^8–13^. In this study, we use a four-center cohort to train and internally test a deep learning model for image-based prediction of *IDH1* mutation status from bone marrow smears (BMS), providing initial proof-of-concept for image-based *IDH1* prediction.

## Methods

### Patient cohorts and image digitization

Patients were eligible upon confirmed diagnosis of AML (excluding acute promyelocytic leukemia) according to WHO/ICC criteria^14,15^, age ≥18 years, available BMS from initial diagnosis, and available information on *IDH1* mutation status detected via next-generation sequencing or targeted sequencing. BMS were prepared from anticoagulated bone marrow and stained using the May-Gruenwald-Giemsa method^16^. 525 whole slide images (WSI) from 436 AML patients from four centers were included in this study. WSI BMS image data were obtained from 191 AML patients from University Hospital Dresden (71 *IDH1* mutated [m], 120 *IDH1* wildtype [wt]; median VAF 43.0, IQR 25.0-46.0), 111 patients from Ohio State University (55 *IDH1*^m^, 56 *IDH1*^wt^; median VAF 27.4, IQR 11.2-43.6), 87 patients from Munich Leukemia Laboratory MLL (38 *IDH1*^m^, 49 *IDH1*^wt^; median VAF 29.0, IQR 15.5-35.3), and 47 patients from LMU University Hospital Munich (22 *IDH1*^m^, 25 *IDH1*^wt^; median VAF 36.9, IQR 35.0-40.9). The *IDH1*^m^ cohort was deliberately enriched for *IDH1*^m^ patients at a roughly equal ratio to *IDH1*^w^ to enable balanced classifier training. Sampling was dichotomized by *IDH* status without restrictions to other class-defining alterations except for *IDH1*^m^ cases excluding *IDH2*^m^. There were no significant differences in median blast counts between *IDH1*^m^ and *IDH1*^wt^ patients (61%, IQR 41.3-80.8% vs. 61%, IQR 38.5-78.0%, *p* = 0.57). WSI scanning was performed at 40x magnification using the Pannoramic Scan II (3DHISTECH, Budapest, Hungary) for both the Dresden and LMU cohorts, at 40x with the Aperio AT2 (Leica, Wetzlar, Germany) for the OSU cohort, and at 60x using the CSFA2000-1000 (Bionovation, Shanghai, China) for the MLL cohort. Images were labeled given the patients’ *IDH1* mutation status as either ‘*IDH1*^m^’ or ‘*IDH1*^wt^’, using a variant allele fraction cut-off of 2%. Prior to analysis, written informed consent was obtained from all patients according to the revised Declaration of Helsinki. All studies were approved by the Institutional Review Board of the Technical University Dresden (EK 98032010) and LMU Munich (204-11). Due to retrospective analysis of de-identified patient data, ethics approval was waived by the IRB at OSU (2023C0066).

### Image preprocessing and deep learning framework

For each WSI, diagnostically relevant regions of interest (ROI) were extracted by generating a low-resolution thumbnail of the WSI and segmenting the image using a customized U-Net model as previously reported^17^, thereby excluding regions with peripheral blood. WSI tile extraction was performed using SlideFlow^18^ at 40 x magnification corresponding to a spatial resolution of 174 × 174 µm. Extracted tiles from ROIs were then ranked using a ResNet^19^-based encoder to obtain low-dimensional feature representations followed by principal component analysis. A set of 387 high-quality tiles was manually curated by two hematologists, evaluating appropriate cellular content, sharp focus, staining quality, and absence of image artifacts. These manually curated high-quality tiles were used to train a Faster R-CNN^20^ architecture to autonomously detect high-quality ROI tiles from all WSIs. The top 40 tiles per patient were retained for downstream analyses.

**Fig. 1:**
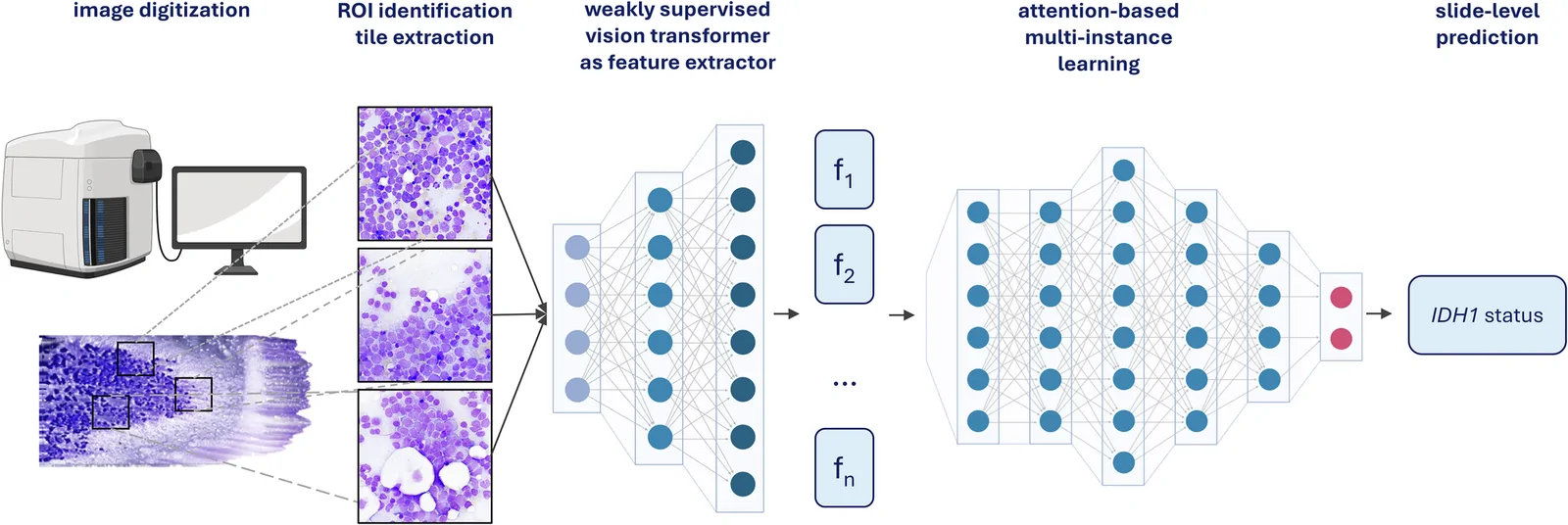
Workflow for *IDH1* mutation status prediction. Bone marrow smears were digitized as whole slide images at 40x magnification. Extracted tiles were used as an input to a weakly supervised vision transformer model that extracted tile-based feature vectors, which were subsequently used as an input to an attention-based multi-instance learning (MIL) model for *IDH1* mutation status prediction at the slide level.

A hierarchical classification framework was then developed for *IDH1* status prediction. Weakly supervised vision transformers were used to extract tile-level feature representations, which were subsequently used as an input for an attention-based multi-instance learning (MIL) model to weigh tiles based on their importance for *IDH1* status prediction and thereby provide a slide-level aggregated prediction (Fig. 1). First, a vision transformer (ViT-Base^21^) was trained in a weakly supervised manner, where slide-level labels (*IDH1*^m^, *IDH1*^wt^) were assigned to individual tiles per WSI. Input tiles were provided at a resolution of 864 × 864 pixels, allowing the model to capture a larger spatial context per tile. Backbone architecture was explored among Vision Transformer variants^21^, specifically ViT-Small and ViT-Base, with patch sizes of 16 and 32. An initial grid search over model variant, patch size, learning rate, and weight decay was performed. ViT-Base at a patch size of 32 was selected for subsequent experiments based on performance. During training, data augmentation was applied, including random horizontal flips, random vertical flips, CutMix augmentation^22^, and MixUp augmention^23^. To strictly avoid data leakage, all tiles belonging to a given patient were assigned exclusively to either the training or validation split, with no overlap between the two. All models were trained using the AdamW optimizer^24^ with cross-entropy loss. Model performance was evaluated using three-fold internal cross-validation with patient-level splits. Each WSI was processed independently and yielded one slide-level prediction. For patients with more than one WSI, slide-level probabilities were averaged into a single patient-level score. All reported metrics were calculated at the patient level, with each patient contributing exactly one prediction. Prediction performance was assessed using macro-averaged receiver operating characteristic area under the curve (ROC-AUC), accuracy, precision, recall, and F1 score, using *IDH1*^*m*^ as the positive class. All metrics are reported as mean ± standard deviation (std) across folds. **Key training parameters are reported in** Supplementary Tables 1, 2.

### Attention-based multiple-instance learning model predicts *IDH1* mutation status from bone marrow smears

**Fig. 2:**
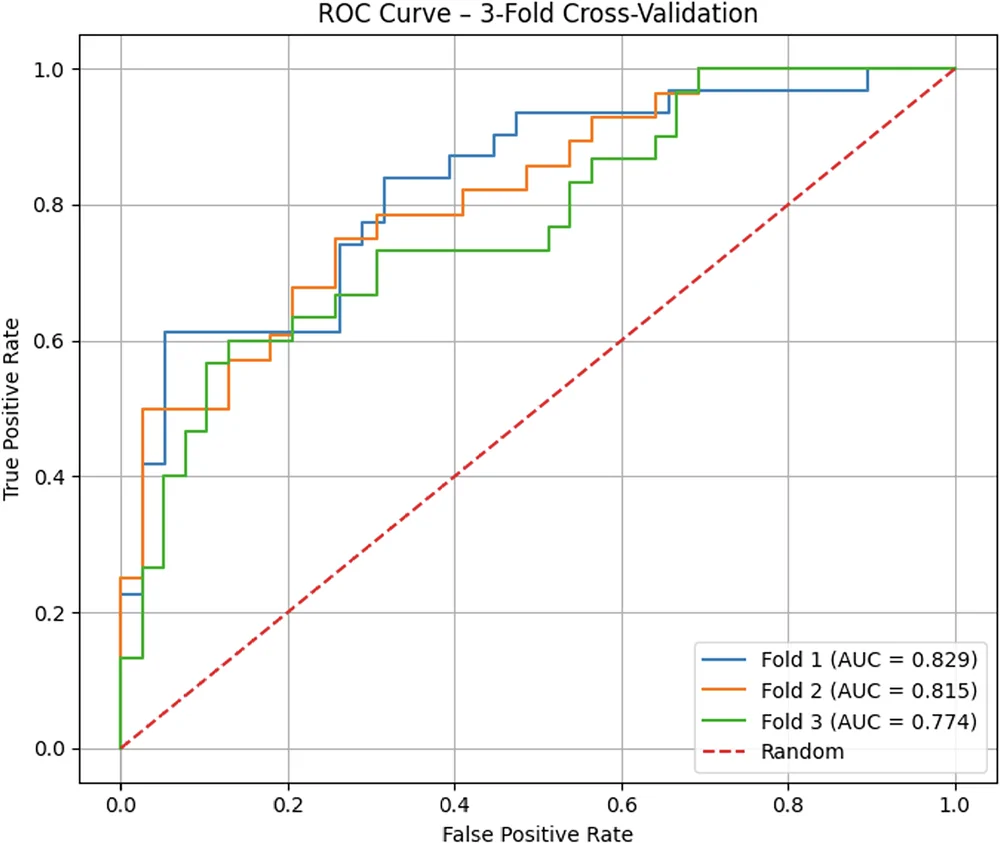
ROC-AUC curves for *IDH1* mutation status prediction with threefold cross-validation using attention-based multi-instance learning.

In preliminary experiments, using only the weakly supervised tile-level classifier, slide-level predictions were obtained by aggregating tile-level probabilities using mean pooling under the assumption that visual cues of *IDH1* status were abundantly present across all tiles. However, this initial approach showed a macro-average F1 score of 0.6769 (std: ± 0.0555), accuracy of 0.7096 (std: ± 0.0258), recall of 0.6867 (std: ± 0.0361), and precision of 0.7542 (std: ±0.0558). We concluded that visual cues informative of *IDH1* status are likely sparse and not present in all tiles equally. To address this limitation, the tile-level weakly supervised classification model trained in the previous stage served as a fixed feature extractor, and an attention-based MIL framework was employed on top for slide-level prediction (Fig. 1). For each tile, the classify (CLS) token embedding from the final transformer layer of ViT-Base was extracted, resulting in a 768-dimensional feature vector per tile. These tile-level feature vectors were then provided as input to the attention-based MIL model, which learns to assign adaptive importance weights to individual tiles based on their relevance for predicting slide-level *IDH1* mutation status. This mechanism enables the model to focus on informative tiles while down-weighting non-discriminative tiles. The attention-based MIL model was also trained using cross-entropy loss and the AdamW optimizer. Hyperparameters were optimized using the Optuna^25^ framework. The attention-based MIL model followed the same patient-level data splits as the tile-level weakly supervised transformer to ensure consistency and prevent information leakage. Attention-based MIL showed a macro-averaged F1 score of 0.7249 (std: ± 0.0210), accuracy of 0.7413 (std: ±0.0231), recall of 0.7223 (std: ±0.0209), and precision of 0.7538 (std: ±0.0471; Supplementary Table 3). Mean ROC-AUC was 0.8057 (95% bootstrap CI, 0.744–0.862) and fold-specific ROC-AUCs were 0.829 (95% CI, 0.725–0.917), 0.815 (95% CI, 0.706–0.908), and 0.774 (95% CI, 0.656–0.877), respectively (Fig. 2). Supplementary Tables 4, 5 provide fold-wise performance metrics and Supplementary Table 6 provides an extrapolation of results at the 8% prevalence of *IDH1*^m^ AML, mimicking real-world deployment. Comparing true positive (TP) predictions with false negative (FN) predictions, we did not find significant differences in mutation type (p.R132c vs. non-p.R132C, Fisher’s exact *p* = 1.00), VAF (TP: median 33,3%; FN: median 39.0%; *p* = 0.27), or median blast counts (TP: 60.8% IQR 40.-81.5%; FN: 68.5% IQR 50.0-79.0%; *p* = 0.53). Explainability methods such as transformer rollout and gradient-weighted class activation mapping (GradCAM) did not show a specific cellular morphology. Reviewing the tiles receiving most attention by the model, we frequently found a lack of mature granulopoiesis in TP specimens, exhibiting more immature phenotypes (Supplementary Fig. 1).

### Clinical implications, limitations, and future directions

Our findings indicate a digital biomarker for *IDH1* mutation status in AML is detectable from bone marrow morphology at the slide level. This is supported by the performance gain of attention-based MIL over tile-wise classification, which suggests subtle morphological changes, e.g., in cellular composition and a lack of maturation. Presently, visual digital biomarkers do not replace genetic testing but may allow for efficient and affordable image-based screening, prompting confirmation with and prioritization of molecular testing as well as supporting diagnostics in low-resource settings. Our study is limited by its retrospective nature and limited sample size, and further (prospective) validation is warranted to evaluate generalizability before clinical translation. To this end, our findings are hypothesis-generating and should not be extrapolated to the general AML population where *IDH1* is much rarer than in our training sample and to other scanners or stains. A larger cohort may also allow adjustment for the co-mutational spectrum of *IDH1*-mutated AML^26^ and, potentially, lead to the development of a unified model of targetable alterations in AML rather than multiple models predicting alterations separately^8–13^. Importantly, any of these models are currently only hypothesis-generating and do not constitute clinical-grade biomarkers. Their merit must be evaluated in prospective clinical trials, demonstrating efficacy and generalizability in multicenter cohorts, and resulting software products have to be in accordance with regulatory requirements prior to routine clinical use.

## Supplementary information

Supplementary Material

## Data Availability

The datasets analyzed during the current study are not publicly available due to data protection and patient confidentiality requirements but are available from the corresponding author on reasonable request.
