# Supplementary material for: Deep learning predicts IDH1 mutation status in acute myeloid leukemia from bone marrow smears
Source: NPJ Precis Oncol. 2026 Oct 29;10:412. doi: 10.1038/s41698-026-01762-x (PMC13720192; doi:10.1038/s41698-026-01762-x)

Supplementary Tables

Supplementary Table 1: Properties of the ViT-B feature extractor

Parameter	Value
Architecture	ViT-Base, patch size 32
Input size	864 × 864 pixels
Pretraining	ImageNet
Optimizer	AdamW
Loss	Cross-entropy
Learning rate	5×10^{-5}
Weight decay	0.05
Batch size	8
Epochs	40
LR schedule	Warm-up and cosine decay
Output	768-dimensional CLS embedding

Supplementary Table 2: MIL Optuna search space used for each of the three folds

Hyperparameter	Search range
Learning rate	1×10^{-5}–5×10^{-4}, log-uniform
Weight decay	1×10^{-6}–1×10^{-3}, log-uniform

Dropout	0.1–0.7
Attention temperature	0.5–2.0
Batch size	8, 16, 32, 64
Trials per fold	30
Optimization objective	Maximize validation F1-score
Training epochs	40

Supplementary Table 3: Cross-validation metrics for attention-based multi-instance learning (MIL) using weakly supervised feature embeddings

**Fold**	**ROC-AUC (macro)**	**Accuracy**	**Precision (macro)**	**Recall (macro)**	**F1 Score (macro)**
Fold 1	0.8285	0.7681	0.8074	0.7479	0.7491
Fold 2	0.8150	0.7313	0.7303	0.7088	0.7129
Fold 3	0.7735	0.7246	0.7236	0.7103	0.7128
Mean ± Std	0.8057 ± 0.0282	0.7413 ± 0.0231	0.7538 ± 0.0471	0.7223 ± 0.0209	0.7249 ± 0.0210

Supplementary Table 4: Fold-wise sample composition and confusion matrix

**Fold**	**n**	***IDH1*^{m}**	**25 *IDH1*^{wt}**	**TN**	**FP**	**FN**	**TP**
Fold 1	69	38	31	36	2	14	17

Fold 2	67	39	28	33	6	12	16
Fold 3	69	39	30	32	7	12	18

Abbreviations: number (n), true negative (TN), false positive (FP), false negative (FN), true positive (TP)

Supplementary Table 5: Fold-wise performance metrics

Fold	ROC-AUC	Accuracy	Sensitivity	Specificity	Observed PPV	Observed NPV
Fold 1	0.8285	76.8%	54.8%	94.7%	89.5%	72.0%
Fold 2	0.8150	73.1%	57.1%	84.6%	72.7%	73.3%
Fold 3	0.7735	72.5%	60.0%	82.1%	72.0%	72.7%

Abbreviations: receiver-operating-characteristic area-under-the-curve (ROC-AUC), positive predictive value (PPV), negative predictive value (NPV)

Supplementary Table 6: Model performance projected to real-world *IDH1* prevalence at 8% of all acute myeloid leukemia cases

Fold	Sensitivity used	Specificity used	Projected PPV at 8%	Projected NPV at 8%
Fold 1	54.8%	94.7%	47.5%	96.0%
Fold 2	57.1%	84.6%	24.4%	95.8%
Fold 3	60.0%	82.1%	22.5%	95.9%

Abbreviations: positive predictive value (PPV), negative predictive value (NPV)

Supplementary Figures

Supplementary Figure 1: Representative test set top tiles

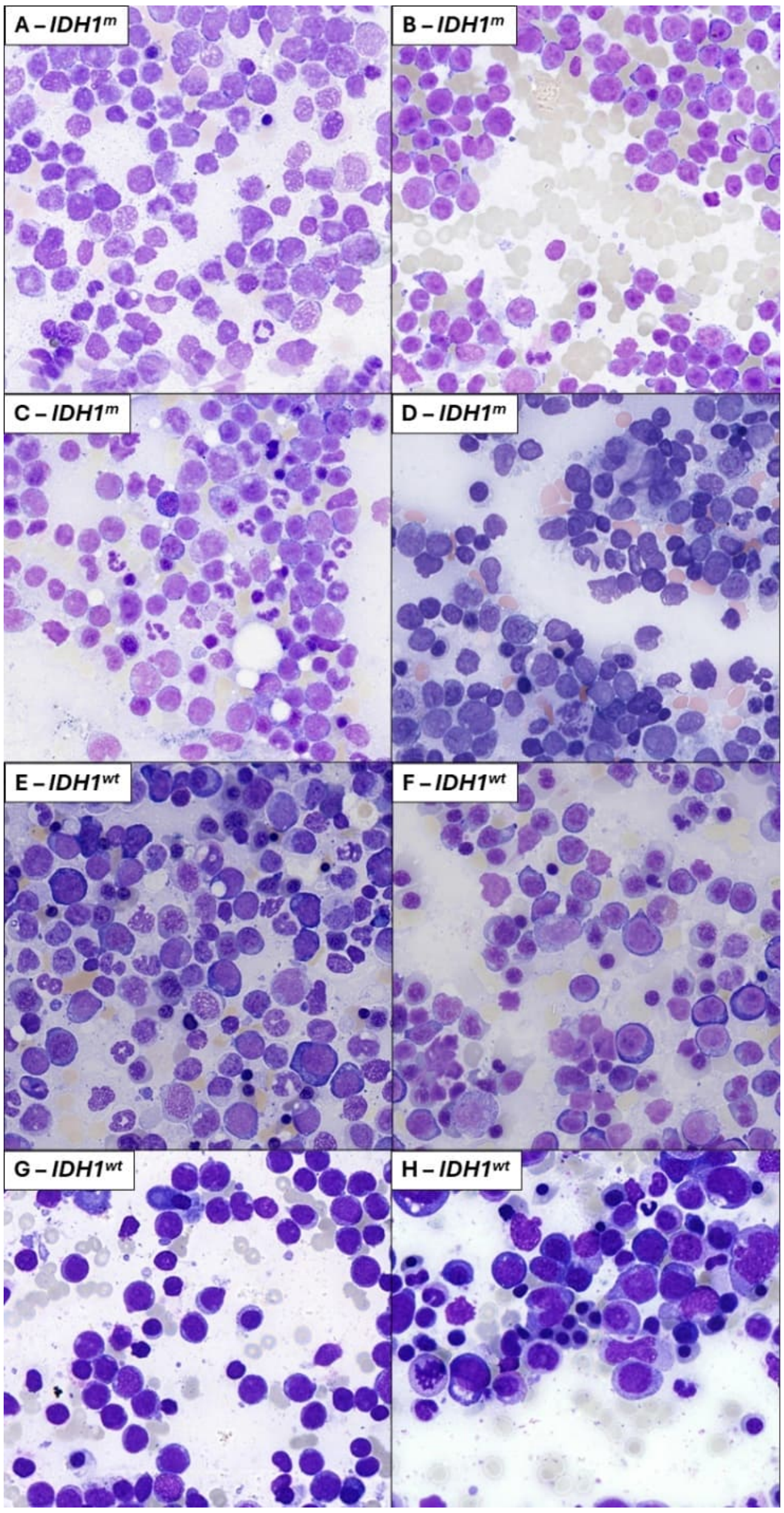

Supplement: Supplementary file 1 [file 41698_2026_1762_MOESM1_ESM.pdf]
